# A Multiparametric Fusion Radiomics Signature Based on Contrast-Enhanced MRI for Predicting Early Recurrence of Hepatocellular Carcinoma

**DOI:** 10.1155/2022/3704987

**Published:** 2022-09-28

**Authors:** Wencui Li, Hongru Shen, Lizhu Han, Jiaxin Liu, Bohan Xiao, Xubin Li, Zhaoxiang Ye

**Affiliations:** ^1^Department of Radiology, Liver Cancer Center, Tianjin Medical University Cancer Institute and Hospital, National Clinical Research Center for Cancer, Tianjin's Clinical Research Center for Cancer, Key Laboratory of Cancer Prevention and Therapy, Tianjin, China; ^2^Tianjin Cancer Institute, National Clinical Research Center for Cancer, Key Laboratory of Cancer Prevention and Therapy, Tianjin, Tianjin Medical University Cancer Institute and Hospital, Tianjin Medical University, Tianjin, China

## Abstract

**Objectives:**

The postoperative early recurrence (ER) rate of hepatocellular carcinoma (HCC) is 50%, and no highly reliable predictive tool has been developed yet. The aim of this study was to develop and validate a predictive model with radiomics analysis based on multiparametric magnetic resonance (MR) images to predict early recurrence of HCC.

**Methods:**

In total, 302 patients (training dataset: *n* = 211; validation dataset: *n* = 91) with pathologically confirmed HCC who underwent preoperative MR imaging were enrolled in this study. Three-dimensional regions of interest of the entire lesion were accessed by manually drawing along the tumor margins on the multiple sequences of MR images. Least absolute shrinkage and selection operator Cox regression were then applied to select ER-related radiomics features and construct radiomics signatures. Univariate analysis and multivariate Cox regression analysis were used to identify the significant clinico-radiological factors and establish a clinico-radiological model. A predictive model of ER incorporating the fusion radiomics signature and clinico-radiological risk factors was constructed. The diagnostic performance and clinical utility of this model were measured by receiver-operating characteristic (ROC), calibration curve, and decision curve analyses.

**Results:**

The fusion radiomics signature consisting of 6 radiomics features achieved good prediction performance (training dataset: AUC = 0.85, validation dataset: AUC = 0.79). The predictive model of ER integrating clinico-radiological risk factors and the fusion radiomics signature improved the prediction efficacy with AUCs of 0.91 and 0.87 in the training and validation datasets, respectively. Furthermore, the nomogram and ER risk stratification system based on the predictive model demonstrated encouraging predictions of the individualized risk of ER and gave three risk groups with low, intermediate, or high risk of ER.

**Conclusions:**

The proposed predictive model incorporating clinico-radiological factors and the fusion radiomics signature derived from multiparametric MR images may be an effective tool for the individualized prediction of postoperative ER in patients with HCC.

## 1. Introduction

Hepatocellular carcinoma (HCC) is one of the most common malignant tumors and ranks as the fourth cause of cancer-related death worldwide [1]. Surgical resection is the most effective curative treatment for HCC patients with well-preserved liver function [2, 3]. However, the clinical outcomes of patients after surgery are poor owing to the 5-year tumor recurrence rate reaching 70%. Mechanisms of tumour recurrence include true recurrence, which mainly manifests as early recurrence, and de novo tumours [4, 5]. Early recurrence (ER) is defined as the detection of intrahepatic and/or extrahepatic HCC recurrence within the first 2 years after surgery. ER accounts for over 70% of tumor recurrence and is also relevant to a worse prognosis [6, 7]. Therefore, developing a highly reliable predictive tool is vital for surgical management, postoperative surveillance, and therapeutic interventions.

Some studies have shown that tumor histological factors, such as poor differentiation grade, microvascular invasion, and satellite nodules, are associated with ER, but these histological features cannot be accessed until postoperation [8–13]. Several HCC staging systems are helpful for prognostic evaluation, reasonable treatment option selection, and clinical research [1], such as the Barcelona Clinic Liver Cancer (BCLC) staging system, Tumor-Node-Metastasis (TNM) staging system, and the Japan Society of Hepatology (JSH) staging system. However, these systems can neither predict ER of HCC nor precisely provide quantitative risk measurements. Novel methods for predicting ER and acquiring more precise prognostic information are urgently needed.

Radiomics is a promising, noninvasive technique that can extract high-dimensional and quantitative imaging features from traditional medical images to provide information about tumor aggressiveness and heterogeneity and predict posttreatment survival of oncology [14, 15]. Some studies on HCC have suggested that radiomics features are useful in predicting microvascular invasion, tumor recurrence, and treatment response [16–18]. To the best of our knowledge, few studies have evaluated the value of predicting postoperative ER of HCC with radiomics analysis based on multiparametric MR imaging to date. Therefore, the purpose of our study was to develop and validate a predictive model of ER of HCC combining radiomics features and clinico-radiological risk factors based on multiparametric MR imaging. Meanwhile, the clinical values of the nomogram and ER risk stratification system based on the predictive model were also investigated.

## 2. Materials and Methods

### 2.1. Study Population

This retrospective study was approved by our institutional ethics committee, and the requirement for informed consent was waived from each patient. The electronic medical records of our institution were reviewed for patients who underwent preoperative abdominal multiparametric MRI and had pathologically confirmed HCC between January 2015 and April 2018. The inclusion criteria of the patients were as follows: (a) multiparametric MR images with good image quality were available; (b) curative hepatectomy was performed within 1 month after MRI examination; and (c) clinico-radiological and follow-up information were complete. The exclusion criteria of the subjects were as follows: (a) the lesion was too small (<1 cm) to draw regions of interest (ROIs); (b) patients with antitumor treatments before surgery, such as repeat liver resection, local ablation, or transarterial chemoembolization; (c) postoperative adjuvant therapy was present; or (d) patients died of other diseases during the follow-up period.

Finally, a total of 302 patients (245 males and 57 females; median age, 5 years; and range, 23–86 years) were included in this study. This study consisted of 141 patients with ER and 161 patients without ER. These patients were divided randomly into a training dataset (*n* = 211; 72 males and 39 females; median age, 57.2 ± 9.8 years; and range, 26–86 years) and a validation dataset (*n* = 91; 73 males and 18 females; median age, 57.6 ± 10.2 years; and range, 23–84 years) at a ratio of 7 : 3.

### 2.2. Follow-Up

All patients were followed up regularly after discharge. Serum *α*-fetoprotein (AFP) levels, liver function tests, and various imaging modalities (ultrasound, contrast-enhanced CT, or MRI) of the abdomen were conducted to monitor recurrence of HCC during follow-up in the first month after liver resection and every 3 or 6 months thereafter [2, 19]. Early recurrence was defined as intrahepatic and/or extrahepatic recurrence of HCC within 2 years after surgery. Recurrence-free survival (RFS) was defined as the interval between the date of surgery and the date of tumor recurrence.

### 2.3. MRI Protocol and Imaging Analysis

A 1.5T MR scanner (Signa Excite HD, GE Healthcare, Milwaukee, WI, USA) with an 8-channel phased-array software coil was used to obtain abdominal multiparametric MR images. MRI sequences included axial in-phase and out-of-phase T1-weighted imaging, axial T2-weighted imaging with fat suppression, and axis diffusion-weighted imaging (DWI). Axial dynamic contrast-enhanced fat-suppressed T1-weighted images (3D Liver Acquisition with Volume Acceleration (3D-LAVA) sequence) were obtained in arterial phases (20–30 seconds), portal venous phases (60–70 seconds), and delayed phases (180 seconds) after bolus injection of 0.1 mmol/kg gadopentetate dimeglumine (Gd-DTPA, Magnevist, Bayer Schering, Berlin, Germany). The details of the MRI parameters are provided in Supplementary Table 1.

All MR images were interpreted independently by 2 radiologists (reader 1 and reader 2, with 4 and 7 years of abdominal MRI experience, resp.) who were aware of HCCs but blinded to clinicopathological parameters and other information. When there was a disagreement, the two radiologists reached final decisions by consensus. The radiologists assessed the following MRI features [20, 21] for each patient: (a) tumor number (0, one lesion; 1, more than one); (b) maximum tumor length (0, *L*-max ≤ 5 cm; 1, *L*-max > 5 cm); (c) tumor margin (0, smooth margin; 1, nonsmooth margin); (d) tumor capsule (0, well-defined tumor capsule; 1, ill-defined tumor capsule); (e) peritumoral enhancement (0, absent; 1, present); (f) rim enhancement (0, absent; 1, present); (g) intratumor necrosis (0, absent; 1, present); (h) intratumor haemorrhage (0, absent; 1, present); (i) two-trait predictor of venous invasion, TTPVI (0, TTPVI-absent; 1, TTPVI-present); (j) peritumoral star nodule (0, absent; 1, present); (k) intratumoral vascularity (0, absent; 1, present); and (l) DWI/T2WI mismatch (0, absent; 1, present). These MRI features of the largest tumor were recorded when the lesions were multifocal.

### 2.4. Clinico-Radiological Risk Factor Selection and Clinico-Radiological Model Development

Routine clinical factors (age, sex, underlying liver disease, Child-Pugh class, histologic differentiation, status of microvascular invasion, etc.) of patients were collected from electronic medical records. The clinical factors and MR characteristics related to ER were selected. The *t*-test or Mann–Whitney *U* test was used to compare continuous variables, and the chi-squared test was used for categorical variables. Univariate analysis was performed to identify significant predictors of ER in the training dataset. Then, those significant factors with a *p*-value less than 0.05 were entered into the multivariate Cox regression analysis. Finally, the clinico-radiological predictive model was constructed in the training dataset with the significant factors of ER (Table 1).

### 2.5. Radiomics Workflow of MR Images

**Figure 1:**
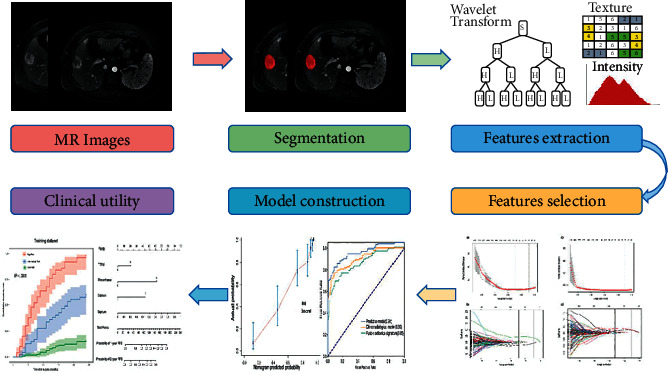
Workflow of radiomics analysis.

The workflow of radiomics consists of tumor segmentation, radiomics feature extraction, radiomics feature selection, radiomics signature construction, model construction, and clinical utility (Figure 1).

### 2.6. Tumor Segmentation and Radiomics Feature Extraction

A radiologist with 4 years of work experience using ITK-SNAP software (version 3.8.0, https://www.itksnap.org/pmwiki/pmwiki.php?n=Main.HomePage) manually drew the region of interest (ROI) of the entire tumor on each transverse slice of T2-weighted images, diffusion-weighted images (*b*-value is 800 s/mm^2^), and arterial phase and portal venous phase images. Twenty tumors were randomly selected and then repeatedly segmented to remove unstable radiomics features, whose intraclass correlation coefficients (ICCs) were lower than 0.80. The Pyradiomics package (version 3.0.0, https://www.radiomics.io/pyradiomics.html) was used to extract 853 radiomics features (16 shape features, 19 first order features, 74 texture features, and 744 wavelet features) from each 3D segmentation. A total of 3412 radiomics features for each tumor (T2WI, DWI, and arterial phase and portal venous phase images) were obtained. The radiomics analysis was carried out by the guidelines of the Image Biomarker Standardisation Initiative [22].

### 2.7. Radiomics Feature Selection and Radiomics Signature Construction

The values of radiomics features extracted from all sequences were normalized with *z*-scores. First, stable radiomics features with ICC values greater than 0.80 were utilized for further analysis (Supplementary Figure 1). Second, the least absolute shrinkage and selection operator was used to select statistically significant radiomics features (Supplementary Table 3, Supplementary Figure 2). Third, the radiomics signature of each MR sequence was constructed with multivariable Cox regression analysis [23]. The formulas of radiomics signatures are provided in the Supplementary Formula. The prediction performances of the radiomics signatures were compared. The final fusion radiomics signature was constructed using radiomics features from the MR sequences, which had a higher performance.

### 2.8. Predictive Model of ER Construction and Evaluation

In the training dataset, the clinico-radiological risk factors and fusion radiomics signature were combined to develop a predictive model of ER with multivariable Cox regression analysis. The discriminative performance of the predictive model was quantitatively evaluated by the area under the curve (AUC) of the receiver operator characteristic (ROC) curve. The accuracy, sensitivity, and specificity were also calculated. A nomogram was developed based on the predictive model of ER. Calibration curves were drawn to evaluate the calibration of the nomogram in the training dataset and validation dataset [24]. The net benefits under different threshold probabilities were quantified by decision curve analysis (DCA) to assess the clinical value of the predictive model [25].

### 2.9. ER Risk Stratification System

A risk stratification system of ER was constructed based on the total score of each individual, which was derived from the predictive nomogram. Its reliability was verified in the validation dataset.

### 2.10. Statistical Analysis

All statistical analyses were performed with SPSS (version 23.0, Chicago, IL, USA) and R software (version 3.6.2, https://www.r-project.org) in this study. Detailed descriptions of the statistical methods and R packages are provided in Supplementary Method 1. A two-tailed *p* < 0.05 was considered statistically significant.

## 3. Results

### 3.1. Clinico-Radiological Characteristics and Clinico-Radiological Model Construction

**Figure 2:**
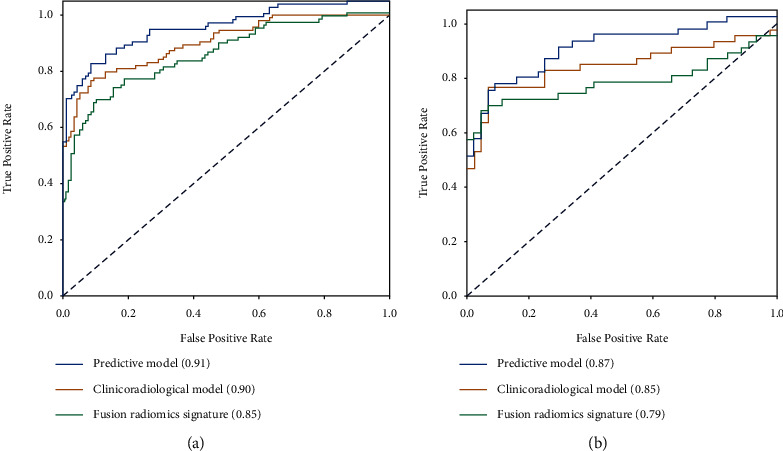
Receiver-operating characteristic curves (ROC) and comparisons of the clinico-radiological model, fusion radiomics signature, and predictive model for the prediction of early recurrence in the training and validation datasets. (a) Training dataset. (b) Validation dataset.

The clinico-radiological characteristics of all patients are shown in Table 1. No significant differences were detected between the training dataset and validation dataset for any of the clinico-radiological factors (*p*=0.146 to 0.976). Univariate analysis showed that aspartate transaminase (AST), *α*-fetoprotein (AFP), *L*-max, tumor margin, tumor capsule, peritumoral enhancement, rim enhancement, TTPVI, intratumor necrosis, intratumor haemorrhage, intratumor vascularity, DWI/T2WI mismatch, histological grade, satellite nodules, and MVI were significantly correlated with ER in the training dataset. All statistical factors were included in multivariable Cox regression analysis to develop the clinico-radiological model. Finally, TTPVI (HR = 3.07, 95% CI: 1.92–4.93, *p* < 0.001), rim enhancement (HR = 4.66, 95% CI: 2.94–7.39, *p* < 0.001), and tumor capsule (HR = 0.38, 95% CI: 0.23–0.61, *p* < 0.001) were predictors of ER. The AUC of the clinico-radiological model was 0.90 (95% CI: 0.83–0.92) in the training dataset and 0.85 (95% CI: 0.76–0.90) in the validation dataset (Figure 2 and Table 2).

### 3.2. Predictive Performances of Radiomics Signatures from Different MR Sequences

The predictive performances of radiomics signatures using MR sequences are shown in Table 2. The radiomics signatures of arterial phase images and portal venous phase images had better predictive performance, with AUCs of 0.85 (95% CI: 0.80–0.91) and 0.81 (95% CI: 0.75–0.87) in the training dataset and AUCs of 0.79 (95% CI: 0.69–0.90) and 0.80 (95% CI: 0.70–0.89) in the validation dataset, respectively. Therefore, arterial and portal venous phase images were used for further analysis. Six radiomics features were selected from all of the stable features of arterial and portal venous phase images to develop the fusion radiomics signature (Supplementary Tables 3 and 4 and Supplementary Formula). The AUCs of the fusion radiomics signature were 0.85 (95% CI: 0.80–0.91) in the training dataset and 0.79 (95% CI: 0.68–0.89) in the validation dataset. The fusion radiomics signature had good predictive efficacy for ER (Table 2).

### 3.3. Predictive Model Construction and Evaluation

**Figure 3:**
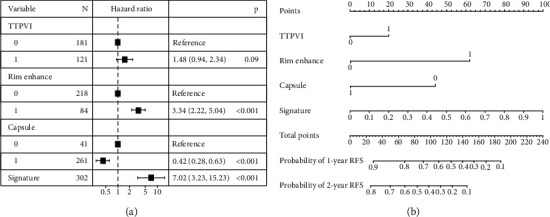
Forest plot and nomogram of predictors of early recurrence. (a) Forest plot of predictors of early recurrence with a multivariate Cox regression model. (b) The model is shown with a nomogram scaled by the proportional regression coefficient of each predictor. (TTPVI: two-trait predictor of venous invasion; rim enhancement; capsule, tumor capsule; signature, fusion radiomics signature).

**Figure 4:**
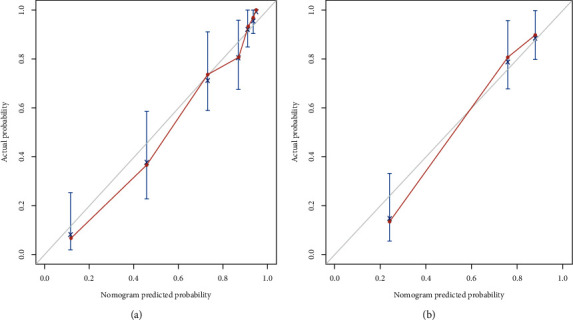
Calibration curves for the training (a) and validation (b) datasets. The *x*-axis typifies the predicted probability of the nomogram for early recurrence, the *y*-axis is the actual early recurrence rate in the patients, and the grey diagonal solid line indicates an ideal prediction by the predictive model.

**Figure 5:**
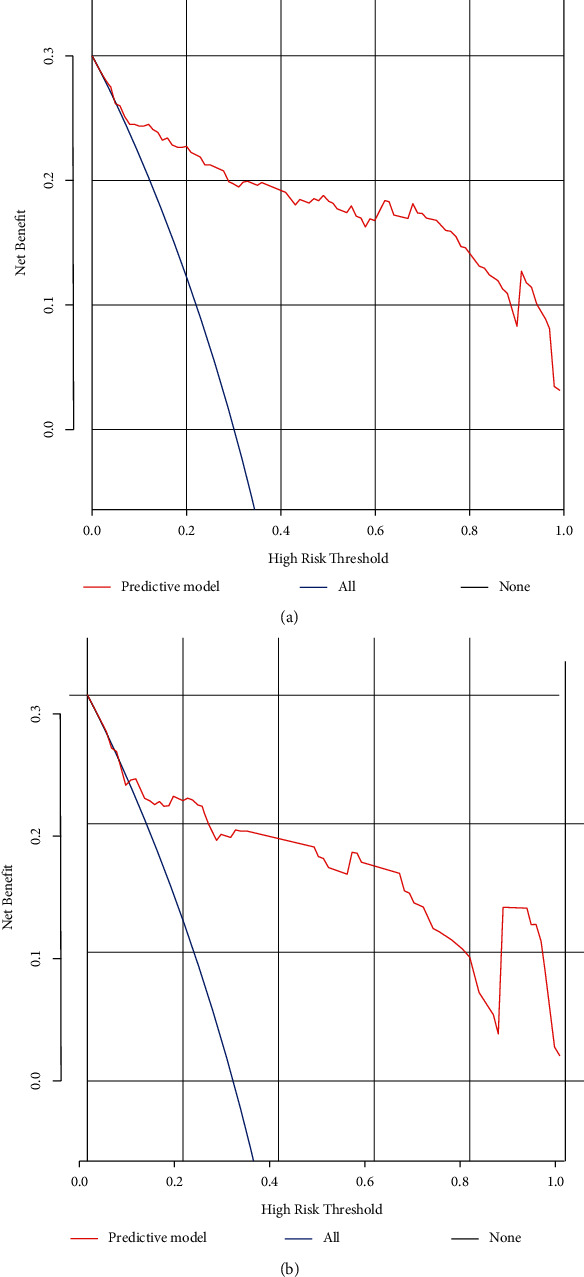
Decision curves for early recurrence in the training (a) and validation datasets. (b) The *y*-axis is the net benefit; the *x*-axis measures the threshold probability. The blue line indicates the net benefit for postulating patients with early recurrence, the black line indicates the net benefit for postulating patients without early recurrence, and the red line represents the expected net benefit of each patient based on the predictive model.

A predictive model of ER that consisted of both clinico-radiological predictive factors and the fusion radiomics signature reached a satisfying performance. For the prediction of ER, the model displayed an AUC of 0.91 (95% CI: 0.87–0.95) with an accuracy, a sensitivity, and a specificity of 85.8%, 91.5%, and 78.7%, respectively, in the training dataset and an AUC of 0.87 (95% CI: 0.79–0.94) with an accuracy, a sensitivity, and a specificity of 81.3%, 88.6%, and 74.5%, respectively, in the validation dataset. The nomogram was developed based on the predictive model of ER, which is presented in Figure 3. The calibration curves of the nomogram (Figure 4) indicated that the predicted probabilities were similar to the actual ER rate in the training and validation datasets. The decision curves showed that the predictive nomogram provided higher net benefits compared with the “treat-all” or “treat-none” scheme within the reasonable threshold probability (Figure 5).

### 3.4. ER Risk Stratification System

**Figure 6:**
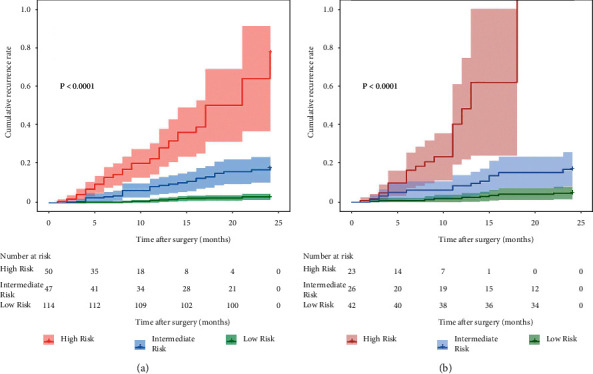
Graphs represent the rates of early recurrence of HCC based on the three risk groups defined by the predictive model in the training and validation datasets.

A risk stratification system of ER was also constructed on the basis of the aggregate score of each individual, which was derived from the predictive nomogram in the training dataset. All patients were divided into low-risk (156/302, 51.6%), intermediate-risk (73/302, 24.2%), and high-risk groups (73/302, 24.2%). In the training and validation datasets, ER rates were 14.9% and 21.4% for the low-risk group, respectively, while they were 59.6% and 57.7% for the intermediate-risk group and 98% and 100% for the high-risk group (Figure 6). Median recurrence-free survival was more than 24 months for the low-risk group, 18 and 15.5 months for the intermediate-risk group, and 7 and 6 months for the high-risk group in the training and validation datasets, respectively (Supplementary Table 2).

## 4. Discussion

In this retrospective work, our research results show that the radiomics signatures can predict postoperative ER of HCC and the prediction model that combines clinico-radiological factors and the fusion radiomics signature has a better predictive efficacy for ER. In addition, the nomogram based on the suggested model shows a good prediction and discrimination ability and could be regarded as a noninvasive tool for the individualized prediction of ER. Furthermore, the risk stratification system developed in our study can successfully separate all of the patients into three distinct ER risk subgroups.

In our study, some of the clinico-radiological factors, such as tumor margin, peritumoral enhancement, intratumor necrosis, microvascular invasion, and AFP, were not significantly correlated with ER of HCC in multivariable Cox regression analysis. These results are inconsistent with those of previous studies [26–28]. The differences might be attributed to the bias of patient selection and different cut-off values of the laboratory factors applied in our and other studies.

ER of HCC occurs in 70% of patients and is also related to a worse prognosis [6, 7]. Therefore, developing a highly reliable predictive tool is important for surgical management, postoperative surveillance, and therapeutic interventions. Radiomics is a novel method that extracts many features from medical images and has been widely used to evaluate the aggressiveness and heterogeneity of HCC [29]. In this study, we developed a predictive model of ER in HCC based on multiparametric MR images with radiomics analysis. Six radiomics features were used to construct the fusion radiomics signature, including five features of arterial phase and one feature of portal venous phase. The performance of the fusion radiomics signature was similar to that of the arterial phase radiomic signature because the fusion radiomics signature was mostly derived from the five radiomics features of the arterial phase images. In addition, we also found that two radiomics features of the arterial phase images were associated with the heterogeneity of ER of HCC: dependence nonuniformity normalized (AP_wavelet.LHH_gldm_DNN) and small dependence emphasis (AP_wavelet.HLL_gldm_SDE). The dependence nonuniformity is usually used to measure the similarity of dependence throughout the image, and the small dependence emphasis is a measure of the distribution of the large dependencies, with a greater value indicative of less homogeneity. These results indicate that radiomics features can offer more information on tumor biological behaviour and the tumor microenvironment, which are complementary to common imaging features. Furthermore, we integrated the fusion radiomics signature and the significant clinico-radiological factors to develop the combined predictive model of ER. The predictive model reached a satisfying performance. Meanwhile, our research results demonstrate that the nomogram and the risk stratification system based on the predictive model are effective tools for the individualized prediction of ER risk and selection of optional treatment and surveillance regimens. For example, patients with a higher risk of ER may be undergoing liver transplantation instead of hepatectomy. If liver transplantation is impossible, appropriate adjuvant treatment and intensive surveillance should be considered [30, 31].

There are several limitations to the present study. One major limitation is that we performed a retrospective study in a single institution; thus, prospective and multicentric studies with considerably large datasets are needed for further validation of the robustness and reproducibility of the present prediction model. In addition, the MRI and radiomics features of the largest tumor were analysed when the lesions were multifocal in our study. Therefore, the results of our study have a certain bias. Third, our study evaluated radiomics features extracted from DWI without apparent diffusion coefficient (ADC) values. The diagnostic performance for the predictive model can be further evaluated when combining the ADC value, one of the quantitative parameters, with other MRI and clinical data. Moreover, manual tumor segmentation was used in our present study, which could cause intra- and inter-user variabilities and can be time-consuming. Thus, semiautomatic and automatic segmentation methods with high accuracy need to be developed.

In conclusion, the prediction model combining clinico-radiological factors and the fusion radiomics signature based on multiparametric MR images achieved an encouraging performance in predicting ER of HCC patients after curative hepatectomy. The nomogram and ER risk stratification system based on the model could have better potential for assisting in clinical decision-making and offering personalized therapies.

## Figures and Tables

**Table 1:** Baseline patient characteristics in the training and validation datasets.

Characteristic	Training dataset (*n* = 211)			Validation dataset (*n* = 91)			*P*-value
	ER (*n* = 94)	Non-ER (*n* = 117)	*P*-value	ER (*n* = 47)	Non-ER (*n* = 44)	*P*-value	
Patient demographics							
Age (y)	57.2 ± 10.3	57.2 ± 9.9	0.976	57.8 ± 10.6	56.2 ± 11.3	0.497	0.976
Gender			0.229			0.226	0.792
Female	14	25		7	11		
Male	80	92		40	33		

Liver disease			0.144			0.363	0.590
Hepatitis B/C virus	56	81		30	32		
Absent	38	36		17	12		

Liver cirrhosis			0.234			0.306	0.418
Present	62	86		33	35		
Absent	32	31		14	9		

Ascites			0.007			0.306	0.641
Present	18	8		7	6		
Absent	76	109		40	38		

Laboratory factors							
ALB (g/l)			0.279			0.967	0.668
≤40	27	26		13	12		
>40	67	91		34	32		

ALT (IU/l)			0.134			0.215	0.292
≤50	70	97		32	35		
>50	24	20		15	9		

AST (IU/l)			0.001			0.024	0.395
≤40	47	86		27	35		
>40	47	31		20	9		

TBIL (*μ*mol/l)			0.349			0.254	0.241
≤19	66	75		37	30		
>19	28	42		10	14		

DBIL (*μ*mol/l)			0.557			0.952	0.974
≤3.4	56	65		27	25		
>3.4	38	52		20	19		

GGT (IU/l)			0.001			0.047	0.173
≤60	37	83		18	26		
>60	57	34		29	18		

AFP (ng/ml)			0.004			0.005	0.781
≤400	55	90		27	37		
>400	39	27		20	7		

CEA (ng/ml)			0.028			0.174	0.610
≤3.4	63	94		29	33		
>3.4	31	23		18	11		

INR	1.04 ± 0.72	1.03 ± 0.87	0.414	1.02 ± 0.08	1.04 ± 0.09	0.316	0.414
PLT (10^9^/l)	183.4 ± 79.3	168.0 ± 64.8	0.119	190.2 ± 70.5	173.3 ± 91.9	0.327	0.447

Child-Pugh grade			0.028			0.791	0.370
A	65	96		33	32		
B	29	21		14	12		

TNM			0.001			0.001	0.796
1	50	98		27	40		
2	11	15		7	2		
3	33	4		13	2		

MRI features							
Multifocality			0.067			0.066	0.486
1	70	99		36	40		
≥2	24	18		11	4		

*L*-max			0.001			0.001	0.186
≤5 cm	31	93		14	32		
>5 cm	63	24		33	12		

Tumor margin			0.002			0.06	0.146
Smooth	12	36		4	10		
Nonsmooth	82	81		43	34		
Tumor-capsule			0.001			0.010	0.813
Present	70	113		36	42		
Absent	24	4		11	2		

Peritumoral enhancement			0.001			0.117	0.660
Present	26	1		8	2		
Absent	68	116		39	42		

Rim enhancement			0.001			0.001	0.847
Present	54	4		25	1		
Absent	40	113		22	43		

TTPVI			0.001			0.001	0.365
Present	63	18		31	9		
Absent	31	99		16	35		

Intratumor necrosis			0.001			0.001	0.773
Present	48	18		25	5		
Absent	46	99		22	39		

Intratumor haemorrhage			0.005			0.051	0.378
Present	22	11		13	5		
Absent	72	106		34	39		

Peritumoral star nodule			0.001			0.001	0.326
Present	27	3		15	2		
Absent	67	114		32	42		

Intratumor vascularity			0.001			0.002	0.324
Hypo-/mild	60	102		27	38		
Hyper	34	15		20	6		

T2WI/DWI mismatch			0.001			0.025	0.210
Present	30	5		9	1		
Absent	64	112		38	43		

Histologic features							
Histologic grade			0.027			0.747	0.412
Well	1	5		1	1		
Moderately	53	81		32	33		
Poorly	40	31		14	10		

Satellite nodules			0.044			0.051	0.905
Present	25	18		13	5		
Absent	69	99		34	39		

MVI			0.015			0.151	0.499
Present	52	45		23	15		
Absent	42	72		24	29		

Data are the number of patients with percentages in parentheses. ALB: serum albumin, ALT: alanine aminotransferase, AST: aspartate aminotransferase, TBIL: total bilirubin, DBIL: direct bilirubin, GGT: *γ*-glutamyl transpeptidase, AFP: *α*-fetoprotein, CEA: carcinoembryonic antigen, INR: international normalized ratio, PLT: platelet count, TNM: tumor-node-metastasis staging system, *L*-max: maximum tumor length, TTPVI: two-trait predictor of venous invasion, and MVI: microvascular invasion.

**Table 2:** The predictive performance of the clinico-radiological model, radiomics signatures using MR sequences and the predictive model.

Different models	Training dataset (*n* = 211)				Validation dataset (*n* = 91)			
	SENS%	SPEC%	ACC%	AUC (95% CI)	SENS%	SPEC%	ACC%	AUC (95% CI)
Clinico-radiological model	80.3	87.2	83.4	0.90 0.83–0.92	77.3	80.9	79.1	0.85 0.76–0.90
T2WI	87.8	65.6	78.0	0.83 0.78–0.89	69.8	67.4	68.5	0.74 0.64–0.85
DWI	94.8	61.3	79.9	0.81 0.75–0.88	88.6	53.2	70.3	0.75 0.65–0.85
Arterial phase signature	89.7	70.2	81.0	0.85 0.80–0.91	88.6	70.2	79.1	0.79 0.69–0.89
Portal venous phase signature	89.7	64.9	78.7	0.81 0.75–0.87	90.9	59.6	74.7	0.80 0.70–0.89
Fusion radiomics signature	89.7	69.1	80.6	0.85 0.80–0.91	90.9	70.2	80.2	0.79 0.68–0.89
Predictive model	91.5	78.7	85.8	0.91 0.87–0.95	88.6	74.5	81.3	0.87 0.79–0.94

A fusion radiomics signature was developed with arterial phase images and portal venous phase images. The predictive model consisted of a fusion radiomics signature and a clinico-radiological model. T2WI: T2-weighted imaging, DWI: diffusion-weighted imaging, SENS: sensitivity, SPEC: specificity, ACC: accuracy, and AUC: area under the curve.

## Data Availability

The data that support the findings of this study are available from the corresponding author upon reasonable request.

## Abbreviations

HCC: Hepatocellular carcinoma
ER: Early recurrence
MR: Magnetic resonance
RFS: Recurrence-free survival
AUC: Area under the curve
ROC: Receiver operator characteristic
DCA: Decision curve analysis.

## Supplementary Materials

Supplementary Materials
*Supplementary Method 1.* The statistical methods and R packages. *Supplementary Table 1*. Imaging parameters of MR sequences. *Supplementary Table 2*. Median recurrence-free survival and cumulative recurrence rates of each risk group defined by the predictive model. *Supplementary Table 3*. Radiomics features after least absolute shrinkage and selection operator. *Supplementary Table 4*. Detailed information of the radiomics features in arterial phase and portal venous phase images. *Supplementary Figure 1*. Histogram of the intraclass correlation coefficient (ICC). *Supplementary Figure 2*. Radiomics features were selected with LASSO Cox regression model. *Method 1*. The statistical methods and R packages. Statistical methods: *t*-test or Mann–Whitney *U* test was used to compare continuous variables, and chi-squared test was used for categorical variables. The univariate analysis was performed to identify significant predictors of ER in the training dataset. All these significant factors with a *p*-value less than 0.05 were entered into the multivariate Cox regression analysis, and the independent significant factors were integrated to construct the predictive model of early recurrence in the training dataset. R packages: the “survival” package was utilized for the multivariable Cox regression model construction. The “caret” package was used to calculate accuracy, sensitivity, and specificity. The ROC curves analysis and calculation of AUC with the “survivalROC” package. The “rms” package was used to develop nomogram and draw calibration curves. The decision curves analysis was performed with “ggDCA” package. Supplementary Formulas: (a) Calculation formula for arterial phase image signature. (b) Calculation formula for portal venous phase image signature. (c) Calculation formula for fusion radiomics signature of arterial phase image and portal venous phase image. The stability of radiomics features were determined with reproducible analysis. Features with an ICC less than 0.8 were excluded; then 410 radiomics features in arterial phase image and 509 radiomics features in portal venous phase image were assessed after robustness test. *Supplementary Figure 2*. Radiomics features were selected with LASSO Cox regression model. (a) Arterial phase image: the partial likelihood deviance curve was drawn versus log (lambda); the optimal lambda value of 0.08705746 (log (lambda) = −2.441187) by using the minimum deviance to select the effective radiomics features. (b) The radiomics features with nonzero coefficients of arterial phase image selected by lambda parameter are shown. (c) Portal venous phase image: the partial likelihood deviance curve was drawn versus log (lambda); the optimal lambda value of 0.07519931 (log (lambda) = −2.587613) by using the minimum deviance to select the effective radiomics features. (d) The radiomics features with nonzero coefficients of portal venous phase image selected by lambda parameter are shown.
