# Supplementary material for: A Multiparametric Fusion Radiomics Signature Based on Contrast-Enhanced MRI for Predicting Early Recurrence of Hepatocellular Carcinoma
Source: J Oncol. 2022 Sep 28;2022:3704987. doi: 10.1155/2022/3704987 (PMC9534653; doi:10.1155/2022/3704987)
Supplement: Supplementary Materials — Supplementary Method 1. The statistical methods and R packages. Supplementary Table 1. Imaging parameters of MR sequences. Supplementary Table 2. Median recurrence-free survival and cumulative recurrence rates of each risk group defined by the predictive model. Supplementary Table 3. Radiomics features after least absolute shrinkage and selection operator. Supplementary Table 4. Detailed information of the radiomics features in arterial phase and portal venous phase images. Supplementary Figure 1. Histogram of the intraclass correlation coefficient (ICC). Supplementary Figure 2. Radiomics features were selected with LASSO Cox regression model. Method 1. The statistical methods and R packages. Statistical methods: t-test or Mann–Whitney U test was used to compare continuous variables, and chi-squared test was used for categorical variables. The univariate analysis was performed to identify significant predictors of ER in the training dataset. All these significant factors with a p-value less than 0.05 were entered into the multivariate Cox regression analysis, and the independent significant factors were integrated to construct the predictive model of early recurrence in the training dataset. R packages: the “survival” package was utilized for the multivariable Cox regression model construction. The “caret” package was used to calculate accuracy, sensitivity, and specificity. The ROC curves analysis and calculation of AUC with the “survivalROC” package. The “rms” package was used to develop nomogram and draw calibration curves. The decision curves analysis was performed with “ggDCA” package. Supplementary Formulas: (a) Calculation formula for arterial phase image signature. (b) Calculation formula for portal venous phase image signature. (c) Calculation formula for fusion radiomics signature of arterial phase image and portal venous phase image. The stability of radiomics features were determined with reproducible analysis. Features with an ICC less than 0.8 were exc [file 3704987.f1.docx]

**Supplementary** **Material**

**Supplementary Material**

Supplementary Method 1. The statistical methods and R packages.

Supplementary Formula.

Supplementary Table1. Imaging parameters of MR sequences.

Supplementary Table2. Median recurrence-free survival and cumulative recurrence rates of each risk group defined by the predictive model.

Supplementary Table 3. Radiomics features after least absolute shrinkage and selection operator.

Supplementary Table 4. Detailed information of the radiomics features in arterial phase and portal venous phase image.

Supplementary Figure 1. Histogram of the intra-class correlation coefficient (ICC).

Supplementary Figure 2. Radiomics features were selected with LASSO Cox

regression model.

**Method 1.** **The statistical methods and R packages**

Statistical methods: t-test or Mann-Whitney U test was used to compare continuous variables, and Chi-square test was used for categorical variables. The univariate analysis was performed to identify significant predictors of ER in the training dataset. All these significant factors with the p value less than 0.05 were entered into the multivariate Cox regression analysis, and the independent significant factors were integrated to construct the predictive model of early recurrence in the training dataset.

R packages: the "survival" package was utilized for the multivariable Cox regression model construction. The "caret" package was used to calculate accuracy, sensitivity, specificity. The ROC curves analysis and calculation of AUC with the "survivalROC " package. The "rms" package was used to develop nomogram and draw calibration curves. The decision curves analysis was performed with "ggDCA" package.

**Supplementary Formula:**

1. **Calculation formula for arterial phase image signature:**

Arterial phase image Signature =

-0.19825308 + 1.1152934 * AP_original_shape_Maximum 3D Diameter +

0.13566372 * AP_wavelet.LHH_gldm_DNN-

0.38115303 * AP_wavelet.HLL_gldm_SDE +

0.07009994 * AP_wavelet.HLH_gldm_SDLGLE-

0.04199232 * AP_wavelet.LLL_glcm_IDN-

0.7979498 * AP_wavelet.LLL_glcm_IMC1

**(b) Calculation formula for portal venous phase image signature:**

Portal venous phase image Signature =

- 0.13723197 + 0.87797874 * PVP_original_shape_Least Axis Length +

0.43634828 * PVP_original_shape_Major Axis Length +

0.05620584 * PVP_original_shape_Maximum 2D Diameter (Column)-

0.13452937 * PVP_ original_glcm_IMC1 +

0.28961985 * PVP_wavelet.LLH_gldm_LDE +

0.0024402 * PVP_wavelet.LLH_glszm_ZE-

0.12558854 * PVP_wavelet.LHH_firstorder_Minimum +

0.07499091 * PVP_wavelet.LHH_gldm_DNN -

0.13226675 * PVP_ wavelet.HLH_gldm_DNN

**(c) Calculation formula for fusion radiomics signature of arterial phase image and portal venous phase image:**

Fusion radiomics signature =

-0.18664596 + 0.81414905 * AP _original_shape_Maximum 3D Diameter +

0.06633864 * AP_wavelet.LHH_gldm_DNN-

0.39691653 * AP_wavelet.HLL_gldm_SDE -

0.09624753 * AP_wavelet.LLL_glcm_IDE -

0.77127915 * AP_wavelet.LLL_glcm_IMC1 +

0.42121636 * PVP_original_shape_Least Axis Length

**Supplementary Figures and Tables**

**Supplementary Table 1** Imaging parameters of MR sequences

| Sequences | TR  (msec) | TE  (msec) | FOV  (mm^2^) | Matrix | Slice thickness  (mm) |
| --- | --- | --- | --- | --- | --- |
| T1WI  (in phase) | 140-170 | 4.2 | 40×34 | 128×128  or 320×160 | 7 |
| T1WI  (out of phase) | 140-170 | 1.8 | 40×34 | 128×128  or 320×160 | 7 |
| T2WI  (fat-saturated sequence) | 7059 | 80 | 40×34 | 128×128  or 320×160 | 7 |
| DWI  (b value is 0  and 800 s/mm^2^) | 4225 | 61.4 | 38-40×  38-40 | 128×128  or 320×160 | 8 |
| Dynamic contrast-enhanced T1WI | 4.7 | 1.7 | 315×316 | 256×256 | 4 |

T1WI=T1-weighted imaging, T2WI=T2-weighted imaging, TR=Repetition time, TE= Echo time, FOV= Field of view; DWI=Diffusion-weighted imaging.

**Supplemental Table2.** Median recurrence-free survival and cumulative recurrence rates of each risk group defined by the predictive model

| Model and group | No.of Patients | Median  RFS (mo) | TRR at  1 Year (%) | ER  Rate (%) | Hazard  Ratio | *P*  Value |
| --- | --- | --- | --- | --- | --- | --- |
| Training dataset |  |  |  |  |  |  |
| High-risk group | 50 | 7 | 68% | 96.0% | 3.36  (2.07, 5.45) ^†^ | 0.000 |
| Intermediate-risk group | 47 | 18 | 34% | 54.5% | 5.50  (3.01, 10.09) ^*^ | 0.000 |
| Low-risk group | 114 | 24 | 6.1% | 14.9% | 1 |  |
| Validation dataset |  |  |  |  |  |  |
| High-risk group | 23 | 6 | 86.9% | 100% | 4.02  (1.94, 8.33) ^†^ | 0.000 |
| Intermediate-risk group | 26 | 15.5 | 34.6% | 57.7% | 3.50  (1.53, 8.02) ^*^ | 0.003 |
| Low-risk group | 42 | 24 | 9.5% | 21.4% | 1 |  |

RFS=recurrence-free survival, TRR=tumor recurrence rate, ER=early recurrence, ^*^Compared with

the low-risk group, ^†^compared with the intermediate-risk group

**Supplementary Table 3.** Radiomics features after least absolute shrinkage and selection operator

| **Sequences** | **T2-weighted image (N=13)** | |
| --- | --- | --- |
| **Features** | T2WI_original_shape_Least Axis Length  T2WI_original_shape_Maximum2D Diameter (Row)  T2WI_original_shape_Maximum3D Diameter  T2WI_wavelet.LHL_glszm_ZE  T2WI_wavelet.LHH_glrlm_LRHGLE  T2WI_wavelet.LHH_glszm_GLN  T2WI_wavelet.LHH_glszm_GLV  T2WI_wavelet.HLL_firstorder_Kurtosis  T2WI_wavelet.HLL_gldm_LDE  T2WI_wavelet.HLL_gldm_SDE  T2WI_wavelet.HLH_firstorder_Kurtosis  T2WI_wavelet.HLH_glszm_GLN  T2WI_wavelet.HHH_firstorder_Kurtosis | |
| **Sequences** | **Diffusion-weighted image (N=7)** | **Arterial phase (N=6)** |
| **Features** | DWI_original_shape_Maximum2D Diameter (Row)  DWI_original_shape_Maximum3D Diameter  DWI_original_gldm_DV  DWI_wavelet.HLL_firstorder_Kurtosis  DWI_wavelet.HLH_ngtdm_Busyness  DWI_wavelet.HHH_gldm_LDLGLE  DWI_wavelet.LLL_ngtdm_Busyness | AP_original_shape_Maximum3D Diameter  AP_wavelet.LHH_gldm_DNN  AP_wavelet.HLL_gldm_SDE  AP_wavelet.HLH_gldm_SDLGLE  AP_wavelet.LLL_glcm_IDN  AP_wavelet.LLL_glcm_IMC1 |
| **Sequences** | **Portal venous phase (N=9)** | **Fusion radiomics signature (N=6)** |
| **Features** | PVP_original_shape_Least Axis Length  PVP_original_shape_Major Axis Length  PVP_original_shape_Maximum2D Diameter (Column)  PVP_original_glcm_IMC1  PVP_wavelet.LLH_gldm_LDE  PVP_wavelet.LLH_glszm_ZE  PVP_wavelet.LHH_first order_Minimum  PVP_wavelet.LHH_gldm_DNN  PVP_wavelet.HLH_gldm_DNN | AP_original_shape_Maximum3D Diameter  AP_wavelet.LHH_gldm_DNN  AP_wavelet.HLL_gldm_SDE  AP_wavelet.LLL_glcm_IDN  AP_wavelet.LLL_glcm_IMC1  PVP_original_shape_LeastAxis Length |

**Supplementary Table 4.** Detail information of the radiomics features in arterial phase and portal venous phase images

| **Feature name** | **Formula** | **Content** |
| --- | --- | --- |
| AP_original_shape_  Maximum 3D  Diameter | Maximum 3D diameter is defined as the largest pairwise Euclidean distance between tumor surface mesh vertices.  Also known as Feret Diameter. | Maximum 3D diameter  is of Shape Features(3D) of the original arterial phase image. |
| AP_wavelet.LHH_  gldm_DNN |  | Dependence Non-Uniformity Normalized of Gray Level Dependence Matrix of arterial phase image transformed  by wavelet filter LHH. |
| AP_waveletHLL_  gldm_SDE | $SDE=\frac{\sum_{i=1}^{N_{g}} \sum_{j=1}^{N_{d}} \frac{p\left(i,j\right)}{i^{2}}}{N_{z}}$ | Small Dependence Emphasis of Gray Level Dependence Matrix of arterial phase image transformed by wavelet filter HLL. |
| AP_wavelet.HLH_  gldm_SDLGLE | $SDLGLE=\frac{\sum_{i=1}^{N_{g}} \sum_{j=1}^{N_{d}} \frac{P\left(i,j\right)}{i^{2}j^{2}}}{N_{\text{z}}}$ | Small Dependence Low Gray  Level Emphasis of Gray Level Dependence Matrix of arterial phase image transformed by wavelet filter HLH. |
| AP_wavelet.LLL_  glcm_IDN | $IDN=\sum_{k=0}^{N_{\text{g}}-1} \frac{\text{p}_{x-y}\left(\text{k}\right)}{1+\left(\frac{\text{k}}{\text{N}_{\text{g}}}\right)}$ | Inverse Difference Normalized of Gray Level Co-occurrence Matrix of arterial phase image transformed by wavelet filter LLL. |
| AP_wavelet.LLL_  glcm_IMC1 | $IMC1=\frac{HXY-HXY1}{\text{max}\left\{HX,HY\right\}}$ | Informational Measure of Correlation 1 of Gray Level Co-occurrence Matrix  Of arterial phase image transformed by wavelet filter LLL. |
| PVP_original_shape_  Least Axis Length | $leastaxis=4\sqrt{\lambda_{least}}$ | Least Axis Length of Shape Features of the original portal venous phase image. |
| PVP_original_shape_  Major Axis Length | $majoraxis=4\sqrt{\lambda_{major}}$ | Major Axis Length of Shape Features of the original portal venous phase image. |
| PVP_original_shape_  Maximum 2D  Diameter (Column) | Maximum 2D diameter (Column) is defined as the largest pairwise Euclidean distance between tumor surface mesh vertices in the row-slice (usually the coronal) plane. | Maximum 2D diameter (Column) of the original portal venous phase image. |
| PVP_original_glcm_  IMC1 | $IMC1=\frac{HXY-HXY1}{\max\left\{HX,HY\right\}}$ | Informational Measure of Correlation 1 of Gray Level Co-occurrence Matrix  Of the original portal venous phase image. |
| PVP_wavelet.LLH_  gldm_LDE | $LDE=\frac{\sum_{i=1}^{N_{g}} \sum_{j=1}^{N_{d}} P\left(i,j\right)j^{2}}{N_{\text{z}}}$ | Large Dependence Emphasis of Gray Level Dependence Matrix of portal venous phase image transformed by wavelet filter LLH. |
| PVP_wavelet.LLH_  glszm_ZE | $\begin{matrix} ZE=-\sum_{i=1}^{N_{\text{g}}} \sum_{j=1}^{N_{s}} p\left(i,j\right)\log_{2}\left(p\left(i,j\right)+\varepsilon\right) \\ \varepsilon\approx2.2\times10^{\text{-}16} \end{matrix}$ | Zone Entropy of Gray Level Size Zone Matrix of portal venous phase image transformed by wavelet filter LLH. |
| PVP_wavelet.LHH_  first order_Minimum | $\text{minimum}=\min\left(\text{X}\right)$ | Minimum of First Order Features of portal venous phase image transformed by wavelet filter LHH. |
| PVP_wavelet.LHH_  gldm_DNN | $DNN=\frac{\sum_{j=1}^{N_{d}} \left(\sum_{i=1}^{N_{g}} P\left(i,j\right)\right)^{2}}{N_{\text{z}}^{2}}$ | Dependence Non-Uniformity Normalized of Gray Level Dependence Matrix of portal venous phase image  Transformed by wavelet filter LHH. |
| PVP_wavelet.HLH_  gldm_DNN | $DNN=\frac{\sum_{j=1}^{N_{d}} \left(\sum_{i=1}^{N_{g}} P\left(i,j\right)\right)^{2}}{N_{z}^{2}}$ | Dependence Non-Uniformity Normalized of Gray Level Dependence Matrix of portal venous phase image transformed  by wavelet filter HLH. |

Where:

LLL, LLH, LHL, LHH, HLL, HLH，HHL，HHH be the intensity value of the transformation images. These transformation images were got from using 8 three-dimensional wavelet filters to process the original image. L is low-pass filter, H is low-pass filter.

**X** be a set of $N_{p}$ voxels included in the ROI

$N_{g}$be the number of discreet intensity values in the image

$N_{d}$ be the number of discreet dependency sizes in the image

$N_{\text{z}}$be the number of dependency zones in the image, which is equal to $\sum_{i=1}^{N_{g}} \sum_{j=1}^{N_{d}} P\left(i,j\right)$

$N_{s}$be the number of discreet zone size in the image

**$P\left(i,j\right)$** be the dependence matrix

$p(i,j)$ be the normalized dependence matrix, defined as$p\left(i,j\right)=\frac{P\left(i,j\right)}{N_{z}}$

$HX=-\sum_{i=1}^{N_{g}} p_{x}\left(i\right)\log_{2}\left(p_{x}\left(i\right)+\varepsilon\right)$ be the entropy of $p_{x}$

$HY=-\sum_{j=1}^{N_{g}} p_{y}\left(j\right)\log_{2}\left(p_{y}\left(j\right)+\varepsilon\right)$ be the entropy of $p_{y}$

$HXY=-\sum_{i=1}^{N_{g}} \sum_{j=1}^{N_{g}} p\left(i,j\right)\log_{2}\left(p\left(i,j\right)+\varepsilon\right)$ be the entropy of $p\left(i,j\right)$

$$HXY1=-\sum_{i=1}^{N_{g}} \sum_{j=1}^{N_{g}} p(i,j)\log_{2}\left(p_{x}\left(i\right)p_{y}\left(j\right)+\varepsilon\right)$$

$P(i,j)$be the zone matrix

$p\left(i,j\right)$be the normalized size zone matrix, defined as $p\left(i,j\right)=\frac{P\left(i,j\right)}{N_{z}}$

**Supplementary Figures**

a. arterial phase b. portal venous phase


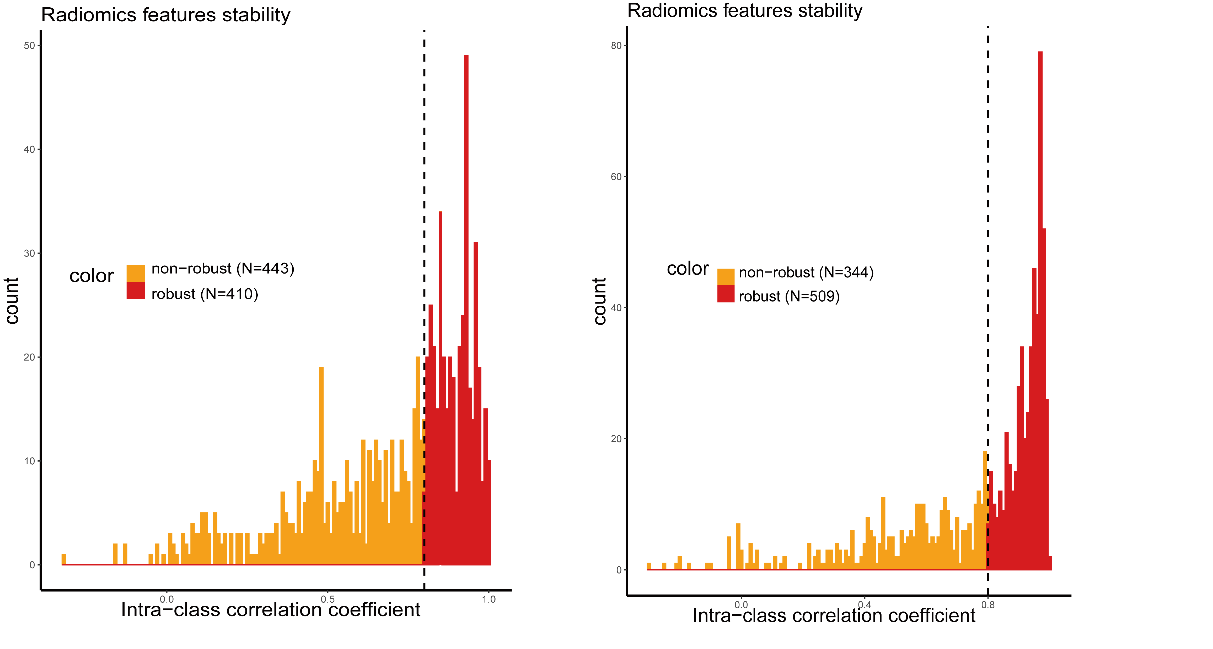


**Supplementary Figure 1**. Histogram of the intra-class correlation coefficient (ICC). The stability of radiomics features was determine with reproducible analysis. Features with an ICC less than 0.8 were excluded, then 410 radiomics features in arterial phase image and 509 radiomics features in portal venous phase image were accessed after robustness test.


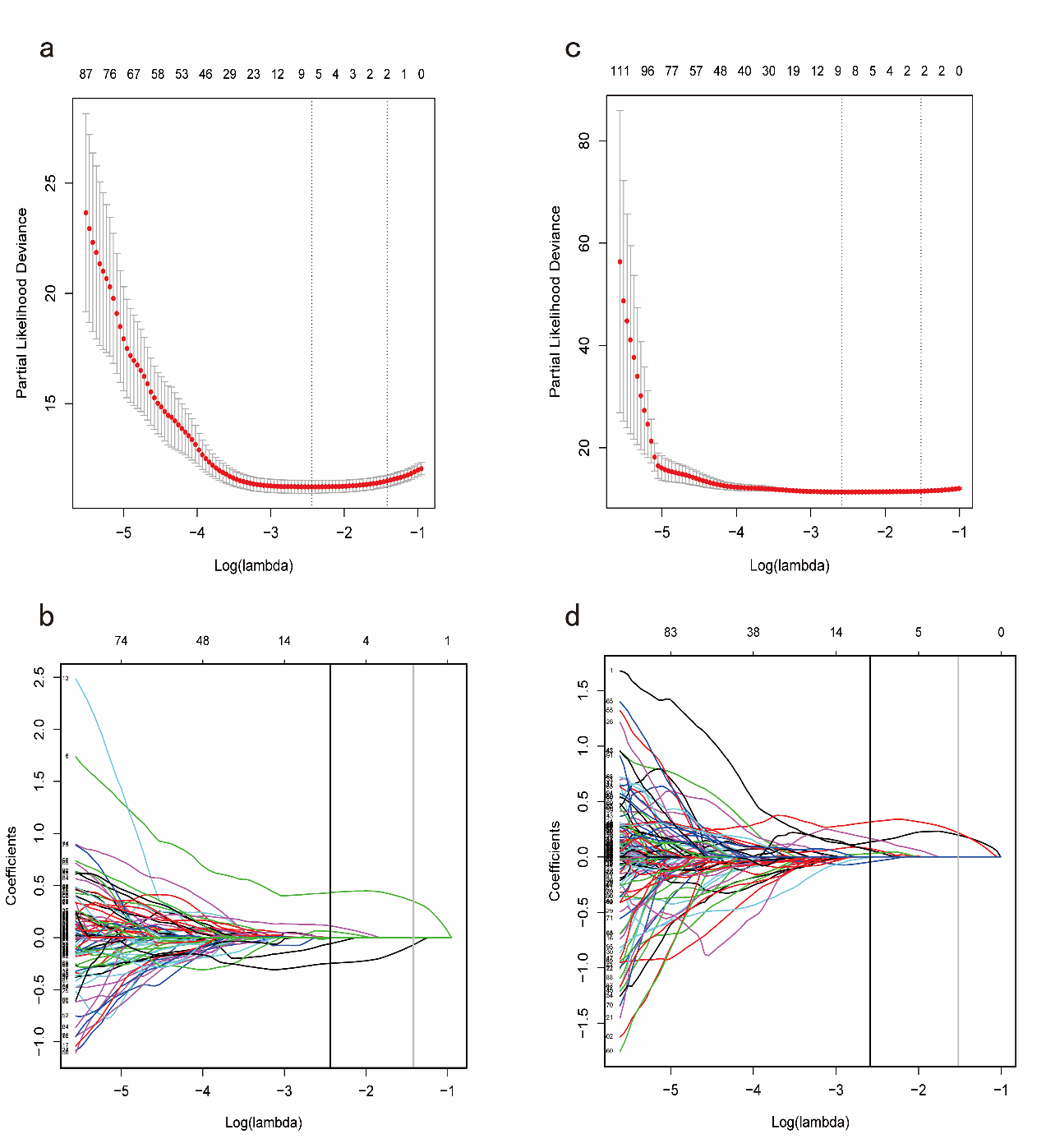


**Supplementary** **Figure 2.** Radiomics features were selected with LASSO Cox regression model. (a) arterial phase image: the partial likelihood deviance curve was drawn versus log(lambda); the optimal lambda value 0.08705746 (log (lambda)= -2.441187) of by using the minimum deviance to select the effective radiomics features. (b) The radiomics features with nonzero coefficients of arterial phase image selected by lambda parameter are shown. (c) portal venous phase image: the partial likelihood deviance curve was drawn versus log (lambda); the optimal lambda value 0.07519931 (log (lambda)= -2.587613) of by using the minimum deviance to select the effective radiomics features. (d) The radiomics features with nonzero coefficients of portal venous phase image selected by lambda parameter are shown.
